# A conceptual framework and protocol for defining clinical decision support objectives applicable to medical specialties

**DOI:** 10.1186/1472-6947-12-93

**Published:** 2012-09-03

**Authors:** Justin W Timbie, Cheryl L Damberg, Eric C Schneider, Douglas S Bell

**Affiliations:** 1 RAND Corporation, 1200 South Hayes Street, Arlington, VA, 22202, USA; 2 Rand Corporation, 1776 Main Street, P.O. Box 2138, Santa Monica, CA, 90407, USA; 3 Rand Corporation, 20 Park Plaza, 9th Floor, Suite 920, Boston, MA, 02116, USA; 4 Rand Corporation, 1776 Main Street, P.O. Box 2138, Santa Monica, CA, 90407, USA

## Abstract

**Background:**

The U.S. Centers for Medicare and Medicaid Services established the Electronic Health Record (EHR) Incentive Program in 2009 to stimulate the adoption of EHRs. One component of the program requires eligible providers to implement clinical decision support (CDS) interventions that can improve performance on one or more quality measures pre-selected for each specialty. Because the unique decision-making challenges and existing HIT capabilities vary widely across specialties, the development of meaningful objectives for CDS within such programs must be supported by deliberative analysis.

**Design:**

We developed a conceptual framework and protocol that combines evidence review with expert opinion to elicit clinically meaningful objectives for CDS directly from specialists. The framework links objectives for CDS to specialty-specific performance gaps while ensuring that a workable set of CDS opportunities are available to providers to address each performance gap. Performance gaps may include those with well-established quality measures but also priorities identified by specialists based on their clinical experience. Moreover, objectives are not constrained to performance gaps with existing CDS technologies, but rather may include those for which CDS tools might reasonably be expected to be developed in the near term, for example, by the beginning of Stage 3 of the EHR Incentive program. The protocol uses a modified Delphi expert panel process to elicit and prioritize CDS meaningful use objectives. Experts first rate the importance of performance gaps, beginning with a candidate list generated through an environmental scan and supplemented through nominations by panelists. For the highest priority performance gaps, panelists then rate the extent to which existing or future CDS interventions, characterized jointly as “CDS opportunities,” might impact each performance gap and the extent to which each CDS opportunity is compatible with specialists’ clinical workflows. The protocol was tested by expert panels representing four clinical specialties: oncology, orthopedic surgery, interventional cardiology, and pediatrics.

## Introduction

Clinical decision support (CDS) is the process of providing persons involved in patient care with intelligently filtered and organized information, at appropriate times, to enable decisions that optimize health care and health outcomes.^a^ The guidance and prompts that CDS can provide constitute one of the primary mechanisms by which electronic health records (EHR) can transform the quality and efficiency of health care delivery [1]. Various studies have demonstrated that CDS can influence clinical practice by helping clinicians to improve diagnosis [2-8], improve quality and patient safety [9-17], adhere to guidelines for prevention and treatment [18-24], and avoid medication errors [25-30].

However, the actual use of CDS within EHRs has been uneven [31,32]. A systematic review of CDS related to medication prescribing cited poor integration of CDS into clinical workflows and limited relevance and timeliness of clinical messaging as two key implementation barriers [33]. The review also found that CDS interventions that were endorsed by colleagues, facilitated doctor-patient interactions, and minimized perceived threats to professional autonomy, were more readily adopted. Other experts have noted that the effectiveness of CDS interventions could be significantly enhanced through clearer displays of relevant information, more effective summaries of vast quantities of information relevant to specific decision making contexts, and by accounting for patients’ comorbidities in clinical recommendations [34]. Many such barriers must be addressed before CDS can truly transform clinical care.

To that end, the Health Information Technology for Economic and Clinical Health Act (HITECH), authorized the Centers for Medicare and Medicaid Services (CMS) to provide incentive payments to eligible providers who successfully demonstrate “meaningful use” of EHRs--including the use of CDS [35]. The initial rules proposed for the EHR Incentive Program included a requirement that providers implement 5 CDS artifacts, each targeted to address one of the specific clinical quality measures that had been designated as options for quality reporting. The final rule required only that providers attest to having implemented one “appropriate” clinical decision support rule that is relevant to the provider’s specialty. The change in approach between the proposed and final rule was responsive to commenters’ concerns that fewer than 5 quality measures would likely be reported by most eligible professionals, and that explicitly linking CDS requirements to clinical quality measures would “put constraints on providers and eliminate many types of CDS that could be beneficial.” [36] Instead, selection of the CDS rule to implement was left to providers, who could take into account their workflow, patient population, and quality improvement efforts. This change was recognized as an interim step taken in the absence of consensus standards for clinically specific CDS requirements. The Stage 2 proposed rule recently reinstated the 5 artifact requirement [37].

The clinical knowledge that should underlie CDS recommendations and the technology available to deliver the knowledge are both rapidly evolving, making it challenging to specify clinically precise meaningful use objectives for CDS. Moreover, performance gaps differ widely across specialties and clinical conditions, making the priorities for the optimal use of CDS potentially distinct within each specialty domain. No framework exists to systematically assess potential CDS objectives to ensure that 1) they address the most critical gaps in care, and 2) they are clinically meaningful to the broad range of specialties participating in the Medicare and Medicaid EHR Incentive Programs.

To begin laying the groundwork for clinically specific standards for high-priority CDS, the Office of the National Coordinator for Health IT (ONC) sought the development of a methodology that would allow experts to define consensus objectives for CDS that would be clinically meaningful within their specialty and that might later be transformed into specific meaningful use objectives for CDS for future stages of the EHR Incentive Program. This paper describes the development of a framework and protocol to elicit high-priority “CDS targets” from panels of specialists, comprising high priority clinical performance gaps within their specialty that are amenable to CDS. We begin by presenting our conceptual framework for specifying high-priority CDS targets. Next, we describe our methodology for identifying candidate clinical performance gaps and CDS opportunities, and the selection, design, and composition of panels for pilot testing the protocol. We then summarize the results of the pilot and the strengths and limitations of the protocol. A detailed report of the study’s methodology and results is available at: http://www.rand.org/pubs/technical_reports/TR1129.html.

## Conceptual framework for specifying high-priority CDS targets

Our approach to defining potential CDS objectives began with a recognition that EHRs could potentially use many kinds of CDS features to target a specific clinical performance gap. For example, the overuse of antibiotics in upper respiratory infections might be targeted by an alert if an antibiotic is prescribed when documentation does not warrant it or by a smart form that guides documentation and therapy selection. In most cases, not only would there be insufficient evidence to determine which CDS intervention is most effective to codify as a specific CDS objective, but furthermore, CDS objectives should be flexible enough to accommodate the rapid pace of innovation in these technologies. On the other hand, meaningful use objectives need to include specific EHR features that can be assessed as present or absent in a given EHR and for which clinician usage can be measured. Given these constraints, we sought to specify CDS “targets,” rather than discrete CDS interventions, that might serve as the basis for future objectives.

**Figure 1:**
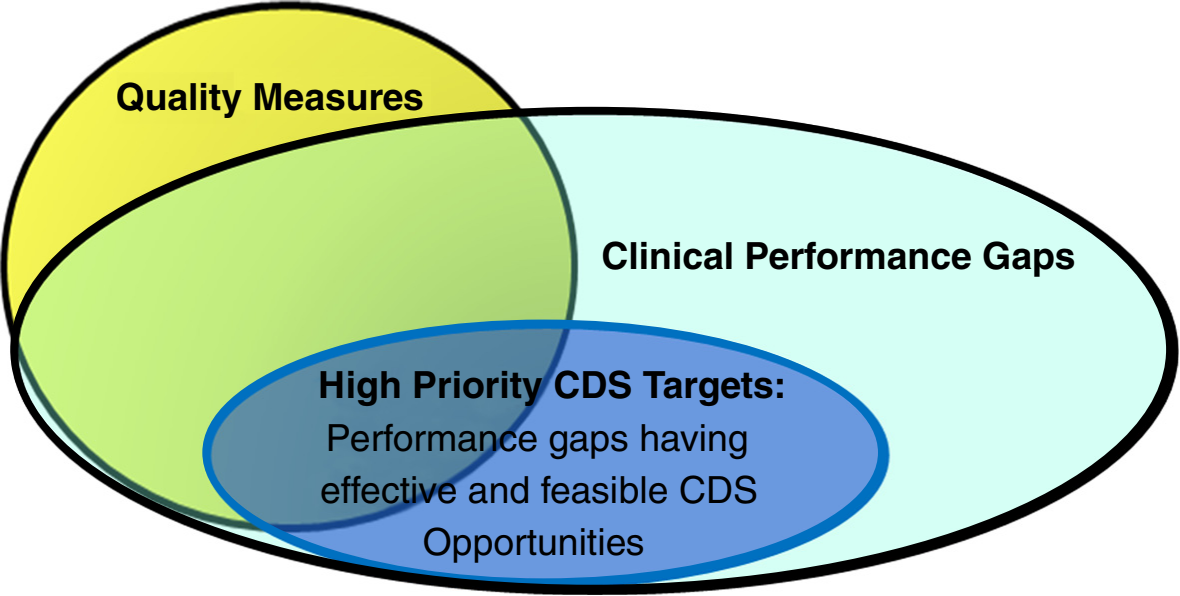
Conceptual framework for defining high priority CDS targets.

We conceptualized a CDS target as a “clinical performance gap” that could be addressed by CDS. Further, we conceptualized **high priority** CDS targets as the most critical clinical performance gaps that have one or more CDS opportunities that can be implemented to address the performance gap that were both effective and compatible with clinical workflow (Figure 1). In the following sections we define clinical performance gaps and CDS opportunities and provide examples of each.

**Clinical Performance Gaps** are clinical areas in which actual practice does not conform to optimal achievable practice. Performance gaps might include:

Failures to deliver care when indicated;

Inappropriate use of diagnostic tests or procedures;

Preventable adverse events;

Disparities or unwanted variations in care delivery; or

Deficiencies in patients’ experience of care (e.g., engagement in decision-making)

A clinical performance gap may or may not be associated with a formal quality measure. Figure 1 illustrates the relationship between quality measures and performance gaps. Although quality measures should always emerge from some recognition of a performance gap, they require many other considerations, including the strength of evidence supporting the desired action, the availability of data to measure each indicator, and the degree to which providers can be held accountable for their performance on the measure. By contrast, the evidence underlying clinical performance gaps may include clinical epidemiology or anecdotal observation in addition to empirical research. Many performance gaps in specialty care do not have associated quality measures because measure development and validation can be a laborious and lengthy process. Thus, as shown in Figure 1, there are many important performance gaps for which quality measures do not exist but which still represent opportunities for improving the quality of care.

### CDS opportunities

A CDS opportunity is a description of a specific CDS intervention, including existing interventions or those that might be developed in the near term, that could be expected to address a clinical performance gap. CDS opportunities might include alerts, order sets, and documentation templates, among other types of interventions. Only a subset of CDS opportunities might be amenable to addressing particular performance gaps (Figure 1), due to either the effectiveness of current CDS technology or to the compatibility of those technologies with the unique aspects of workflow within the specialty.

The main task of the panel was to consider the importance of an initial set of performance gaps, and then to consider the strength of the CDS opportunities for the highest rated gaps. “High priority CDS targets” were those performance gaps that were both rated highly important and for which the CDS opportunities to close the gap were rated as having high potential impact and being highly compatible with clinical workflows. Table 1 provides a sample of clinical performance gaps from each of the four specialties and an associated CDS opportunity.

**Table 1:** Four illustrative CDS targets: clinical performance gaps and CDS opportunities

**Clinical Performance Gap**	**CDS opportunity targeting the gap**
Many patients receiving chemotherapy are at risk of experiencing adverse events due to errors in chemotherapy ordering. [Oncology]	Alert at the time of ordering or infusion if chemotherapy orders differ from accepted standards
Patients undergoing total hip or total knee replacement surgery may not receive VTE prophylaxis when it is indicated. [Orthopedic surgery]	Order set for VTE prophylaxis that recommends treatment customized to patient’s bleeding risk and that conforms to guidelines
Nearly half of patients with STEMI receive no reperfusion therapy or receive delayed reperfusion (>12 hours after onset). [Interventional cardiology]	Alert to inform ED physician and staff of possible ACS diagnosis triggered by abnormal biomarkers
Children with asthma are not routinely monitored for control of their condition. [Pediatrics]	Pathway to guide dose escalation or medication substitution

Notes: VTE = Venous Thromboembolism, STEMI = ST Segment Elevation Myocardial infusion, ED = Emergency Department, ACS = Acute Coronary Syndrome.

## Methodology for identifying candidate clinical performance gaps and CDS opportunities

In preparation for each expert panel, study staff worked with the panel chair and co-chair to identify candidate clinical performance gaps and CDS opportunities from a wide range of sources.

### Clinical performance gaps

We used three approaches to identify candidate clinical performance gaps for each panel. First, we scanned existing quality measure repositories and websites of quality measure producers, including the National Quality Measures Clearinghouse, National Quality Forum, Physician Quality Reporting System, National Committee for Quality Assurance, American Medical Association Physician Consortium for Performance Improvement, Hospital Inpatient Quality Reporting Program, Premier Hospital Quality Incentive Demonstration, and the quality measures selected for reporting in the Stage 1 proposed rule for the Medicare and Medicaid EHR Incentive Programs. Quality measures selected as relevant for each specialty were restated as declarative gap statements (Table 1). Second, we conducted an environmental scan to collect data, where available, on the prevalence of each performance gap and the clinical and economic outcomes of each gap—including both morbidity and mortality if appropriate—to provide panelists with a source of objective information about the relative importance of each gap prior to the ratings. Finally, we reviewed the preliminary list of performance gaps with the panel co-chairs, who recommended additions and/or revisions to the list. During the panel’s first meeting, we asked panelists to nominate additional performance gaps, which were included in the rating process. Throughout, we sought to represent gaps from each of the six priority domains of the National Priorities Partnership [38] — patient and family engagement, population health, safety, care coordination, palliative and end-of-life care, and elimination of overuse.

### CDS opportunities

**Figure 2:**
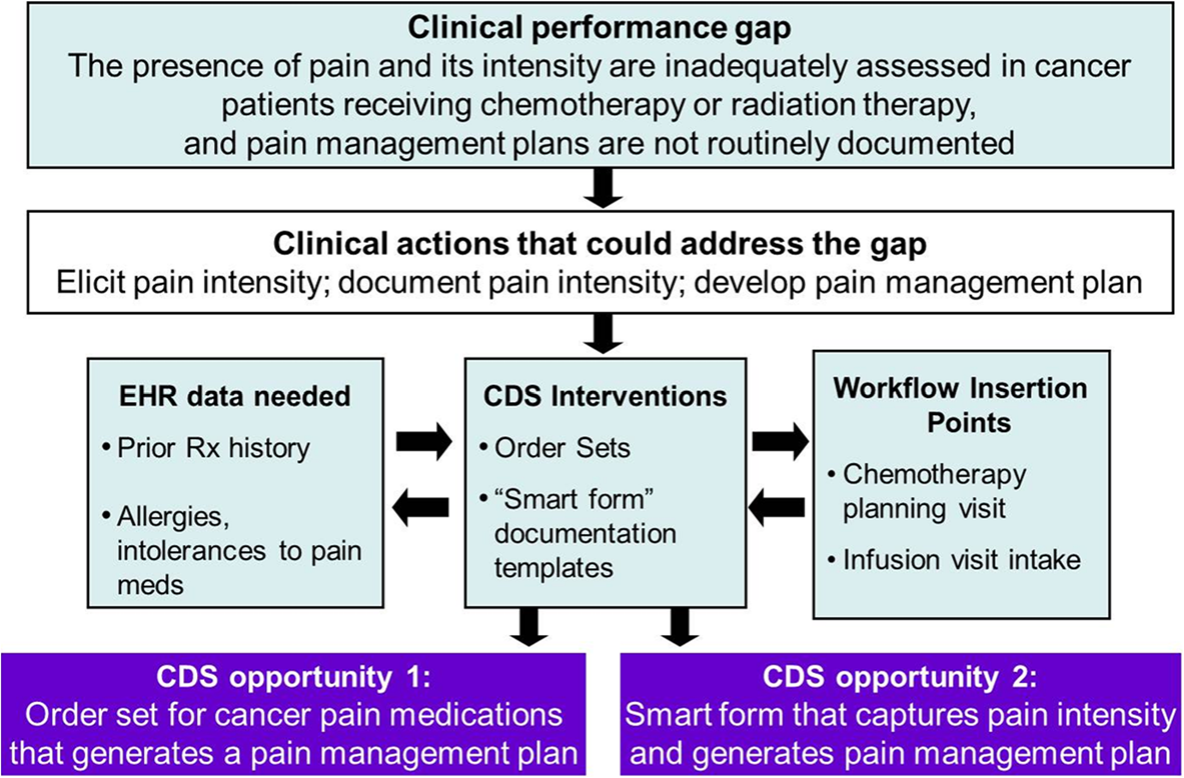
Approach for specifying CDS opportunities for clinical performance gaps.

We then identified CDS opportunities for each of the clinical performance gaps using an approach depicted in Figure 2. First, for each performance gap, we identified one or more clinical actions that physicians and other health care professionals could take to address the clinical performance gap based on team members’ clinical experience. Second, we considered how specific CDS interventions could support providers in taking those clinical actions. These choices were influenced by two other factors: 1) the type of information that would be needed by (and available to) the CDS tool to be able to support the clinical action, and 2) consideration of the clinical workflow into which the tool might be inserted. For each specialty area, we conducted a scan of the published literature for specific CDS interventions that had been used within the specialty domain, and abstracted data on their effectiveness and their impact on workflow. We were unable to collect data on CDS tools in development or those for which evaluations were not published in the peer-reviewed literature.

To supplement the tools described in the literature, we worked with the panel co-chairs to identify additional CDS interventions. We sought to identify both existing tools and CDS concepts—assuming that there would be adequate lead time to allow vendors to develop these tools before later stages of meaningful use objectives were released. Although we did not provide panelists with an opportunity to add candidate CDS opportunities because of time constraints, such an approach would be desirable.

We also sought to identify CDS opportunities that covered a broad range of CDS categories. We used a taxonomy developed by Osheroff and colleagues [39] that included six categories of CDS interventions (Table 2).

**Table 2:** CDS intervention categories with examples

**Category**	**Examples**
Documentation forms and templates	Clinician encounter documentation forms; patient self-assessment forms; “smart forms;” data flow sheets
Relevant data presentation	Relevant data for ordering, administration, or documentation; retrospective/aggregate reporting or filtering; choice lists; practice status display
Order/prescription creation facilitators	Order sets; tools for complex ordering
Protocol and pathway support	Stepwise processing of multistep protocol or guideline; support for managing clinical problems over long periods and many encounters
Reference information and guidance	Context-specific direct links to specific, pertinent reference information
Alerts and reminders	Alerts to prevent potential/omission/commission errors or hazards; alerts to foster best care

Note: A “smart form” is an EHR-based clinical workflow tool designed for organized data review for specific conditions, effective and efficient facilitated data capture, documentation of a clinical visit, and integrated, dynamic, actionable decision support in a single environment [40].

In Table 3, we provide examples of the workflow elements that guided the development of CDS opportunities. No comprehensive workflow framework was available to draw on to select optimal insertion points in workflows. Moreover, different clinicians or practice organizations may insert different forms of CDS into different workflows, and so a one-size-fits all approach may not be appropriate. The framework we developed for the purposes of this project decomposes workflow into specific tasks, the actors or persons who take action, and the settings in which the task might occur.

**Table 3:** Examples of workflow elements

**Tasks**	**Actors**	**Settings**
Prescribing	· Specialist	· Office
Ordering a test	· Physicians’ assistant	· Ambulatory clinic
Gathering clinical data from a patient	· Nurse	· Hospital
Interpreting a test result	· Advanced practice registered nurse	· Emergency department
Generating a note or consult report	· Administrative assistant	· Ambulatory surgery center
Receiving a consult report	· Visiting nurse	· Patient web portal
Communicating results to a patient	· Patient, family, or caregiver	
Discharging a patient		

## Expert panel protocol

### Meeting format and rating process

**Figure 3:**
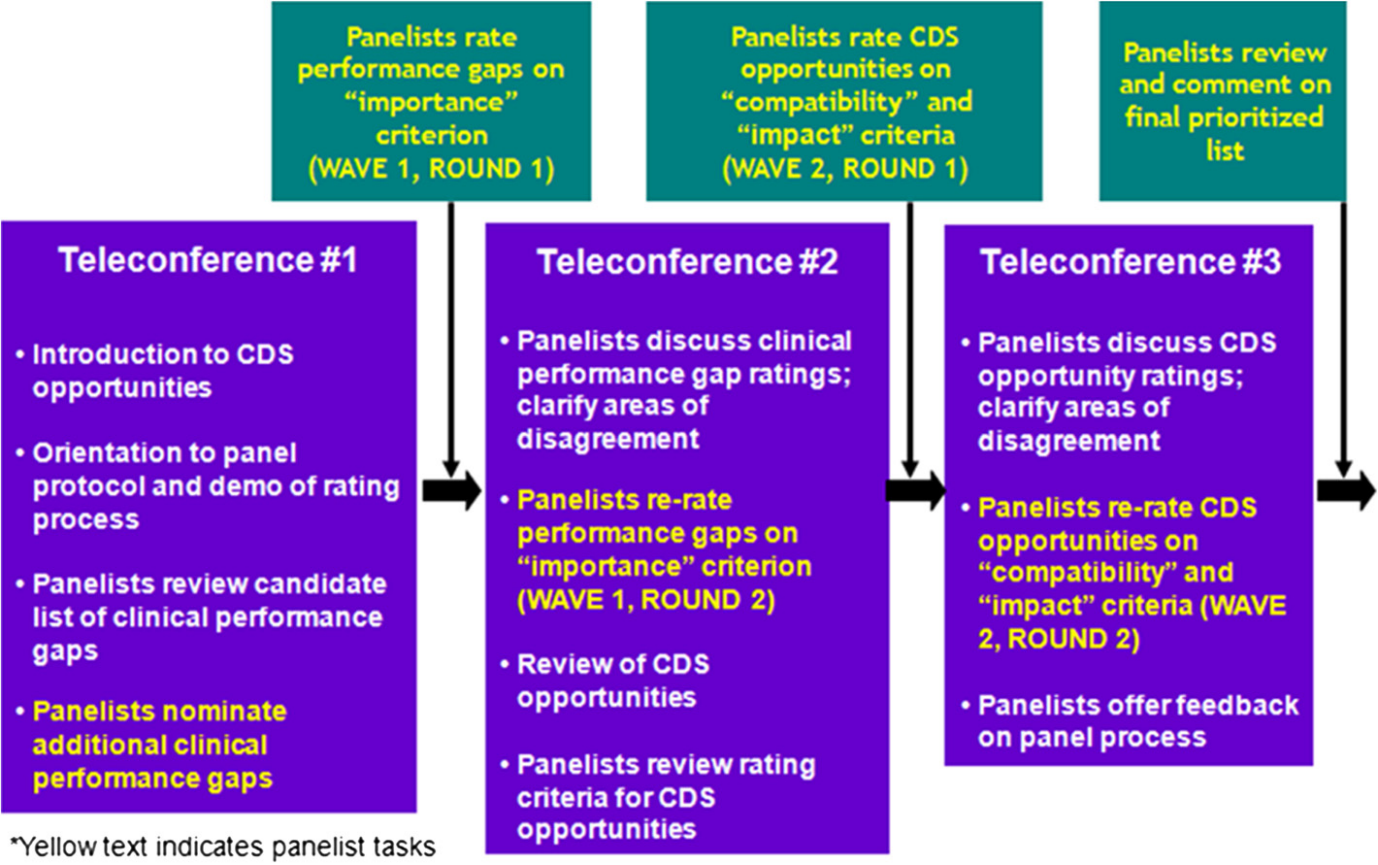
Expert panel protocol.

We used a teleconference meeting format with webinar, hosting three 90-minute teleconferences with each panel (Figure 3). Across these three meetings the panelists completed two modified Delphi rating processes, one focused on rating the **importance** of each performance gap, and the second focused on rating, for each important performance gap, the *compatibility* of CDS with clinical workflow and the *potential impact* for CDS to close the performance gap. Each rating process began with an initial round of ratings that the panelists conducted independently and confidentially. Panelist ratings were then compiled for review and discussion on a panel teleconference, and the discussion was then followed immediately by a second round of ratings, which the panelists were asked to complete before leaving the call. Panelists submitted their ratings electronically to facilitate data collection, ensure completeness of data, and to expedite the analysis.

Panelists received a summary of the first round of ratings prior to attending the second and third calls to allow them to review their ratings relative to those of other panelists. Table 4 shows a one page example of a report from the first round of ratings of the oncology CDS opportunities. Panelists could see the distribution of initial ratings by looking at the numbers above the 1 to 9 rating line, which shows counts of the number of panelists who selected each value. For example, 2 panelists assigned a rating of “6” for the *compatibility* of the first CDS opportunity for addressing Gap #3 (a smart form that captures pain intensity). Each panelist received a different printout; the distribution of ratings was the same on all reports, but the caret (^) below the rating line showed the initial rating assigned by an individual panelist.

**Table 4:** Sample panelist rating report depicting the distribution of panelists’ ratings and the panelist’s own rating

**Oncology Gaps/CDS Opportunities**	**Compatibility**									**Potential Impact**								
**Gap #3:** The presence of pain and its intensity are inadequately assessed or quantified in cancer patients receiving chemotherapy or radiation therapy, and pain management plans are not routinely documented.																		
Smart form that captures pain intensity and generates pain management plan based on patient preference and particular history			**1**		**1**	**2**	**5**	**3**					**1**		**3**	**3**	**4**	**1**
	**1**	**2**	**3**	**4**	**5**	**6**	**7**	**8**	**9**	**1**	**2**	**3**	**4**	**5**	**6**	**7**	**8**	**9**
						**^**									**^**			
Display cancer pain history with intensity levels and current/prior treatments for pain				**1**	**2**	**1**	**1**	**6**	**1**			**1**	**2**		**2**	**3**	**2**	**2**
	**1**	**2**	**3**	**4**	**5**	**6**	**7**	**8**	**9**	**1**	**2**	**3**	**4**	**5**	**6**	**7**	**8**	**9**
								^									^	
Order set for cancer pain medication that results in a comprehensive management plan						**3**	**3**	**4**	**2**						**1**	**3**	**6**	**2**
	**1**	**2**	**3**	**4**	**5**	**6**	**7**	**8**	**9**	**1**	**2**	**3**	**4**	**5**	**6**	**7**	**8**	**9**
							^											^
Pathway to guide initial selection of pain medication and to guide escalation of therapy when required						**2**	**4**	**3**	**3**				**1**		**1**	**4**	**5**	**1**
	**1**	**2**	**3**	**4**	**5**	**6**	**7**	**8**	**9**	**1**	**2**	**3**	**4**	**5**	**6**	**7**	**8**	**9**
						^											^	
Reminders to assess and to quantify pain at appropriate moments in workflow		**1**				**1**	**3**	**6**	**1**			**1**			**1**	**6**	**4**	
	**1**	**2**	**3**	**4**	**5**	**6**	**7**	**8**	**9**	**1**	**2**	**3**	**4**	**5**	**6**	**7**	**8**	**9**
						^									^			
Overall rating					**1**	**1**	**5**	**4**	**1**			**1**			**1**	**3**	**6**	**1**
	**1**	**2**	**3**	**4**	**5**	**6**	**7**	**8**	**9**	**1**	**2**	**3**	**4**	**5**	**6**	**7**	**8**	**9**
							^										^	

Note: The figure displays a portion of a rating report provided to Panelist #13. The distribution of all panelists’ ratings are depicted in the row directly above the rating scale. Each panelist’s own rating is indicated by a caret. For example, two panelists (including Panelist #13) assigned a rating of 6 for the compatibility of the first CDS opportunity, a smart form that captures pain intensity.

During the second and third teleconferences, the discussion of first round gap and CDS opportunity ratings was led by a clinician who was involved in developing the initial list of gap statements and associated CDS opportunities. The discussion during the second and third teleconferences focused on those items that, after analysis of the first round of ratings, were associated with “indeterminate” levels of agreement based on the dispersion in panelists’ ratings (See Analysis of Ratings, below). After discussion, the panelists were then asked to independently and confidentially re-rate all of the gaps (during teleconference #2) and gap-CDS opportunity pairs both individually and as a set of opportunities for a given gap statement (during teleconference #3).

### Rating criteria

We developed three criteria for panelists to use to rate individual performance gaps and CDS opportunities (Tables 5 and 6). In defining the *importance* criterion, we looked to the National Priorities Partnership framework to identify potential dimensions of importance, from which we selected three: 1) population health (i.e., prevalence, health impact on individuals), 2) patient engagement, and 3) efficiency. We also included an additional dimension, the extent to which evidence supports specific scientific actions to address each performance gap. In assessing *compatibility*, panelists were instructed to consider a range of practice workflows into which the CDS intervention might be inserted including their own workflows, those of ancillary staff, and those that might be common in other practice settings. This criterion was considered important because the timing with which CDS is introduced in a workflow and its level of intrusiveness could impact the overall utility of the tool. In assessing the *potential impact* of CDS on the performance gap, panelists were asked to imagine how CDS tools would promote actions to address each gap. Panelists used a nine-point scale with the following anchors: for *performance gap* ratings (1 = Not at all important; 5 = Equivocal; 9 = Extremely important)) and for *CDS opportunities* (1 = Not at all compatible/No potential impact; 5 = Equivocal; 9 = Extremely compatible/Extremely high potential impact).

**Table 5:** Rating criteria used to elicit CDS priority performance gap-CDS opportunities: clinical performance gaps

	
Criterion 1: Importance	· Affects a relatively large number of patients (prevalence)
	· On average, there are significant consequences to the patient in terms of increased risk of morbidity or mortality
	· The gap may be addressed by patient engagement and delivery of more patient-centered care
	· Poor performance leads to inefficient use of resources/waste in health care spending
	· Scientific evidence or professional consensus exists on one or more actions to address the performance gap

**Table 6:** Rating criteria used to elicit CDS priority performance gap-CDS opportunities: CDS opportunities

	
Criterion 1: Compatibility of CDS with workflow	· One or more of the CDS tools within the opportunity set can be readily introduced into a specialist’s workflow and/or the workflow of others on the care team
	· The specialist or other members of the care team are likely to use the CDS tools in daily practice
Criterion 2: Potential impact of CDS on the performance gap	· Information deficiencies or low-reliability systems are the main contributor to the performance gap rather than clinical uncertainty, insufficient scientific evidence, or other factors
	· The CDS tool can provide the majority of the information needed to address the clinical gap

## Analysis of ratings

Following conventional methods for analyzing data from a modified Delphi process [41], we computed three sets of estimates for each performance gap and CDS opportunity. First, we calculated *median* ratings to measure the central tendency for the set of panelists’ ratings. We then estimated *mean absolute deviations* from the median to measure the dispersion of the ratings. Third, we classified ratings as exhibiting agreement, disagreement, or indeterminate levels of agreement, using nonparametric decision rules that take into account the distribution of panelists’ scores. “Agreement” is achieved when panelists’ ratings converge tightly around the median rating, while “disagreement” reflects a polarization of opinion that occurs when a large number of panelists provide ratings in both extremes of the rating scale. Because typical rules for measuring agreement are based on panels of size 9 and our panels ranged in size from a low of 12 to a high of 17, we followed the generalized scoring method identified in the RAND/UCLA Appropriateness Method manual [41] for measuring *disagreement* and *agreement* with larger panels, as shown in Table 7.

**Table 7:** Definitions of agreement and disagreement for different panel sizes

	**Agreement**	**Disagreement**
**Panel size**	**Number of panelists rating outside the 3-point region containing the median**	**Number of panelists rating in each extreme third of the scale (1-3 and 7-9)**
8-10	≤2	≥3
11-13	≤3	≥4
14-16	≤4	≥5

Note. Definitions of disagreement and agreement reflect the default definitions according to the developers of the RAND/UCLA Appropriateness Method. These are also the most widely used definitions.

According to the RAND/UCLA Appropriateness Method, classification of results depends only on median ratings and the presence or absence of disagreement. Performance gaps with median ratings in the top third of the 9-point scale without disagreement are classified as *important*, those with median ratings in the bottom third without disagreement are classified as *unimportant*, and those with intermediate median ratings or any median with disagreement are *equivocal* (Table 8). However, we used more restrictive criteria to identify high priority clinical performance gaps, by requiring that each gap have both a median rating between 7 and 9 and exhibit statistical agreement. Items with indeterminate levels of agreement were not considered high priority. A similar procedure was used in rating the CDS opportunities for the high priority gaps.

**Table 8:** Classification of performance gaps and CDS opportunities based on median ratings and statistical agreement, by rating criterion

	**Rating Criterion**	**Rating result**			
		Median: 1-3 AND No Disagreement*	**Median: 4-6 OR Disagreement**	**Median: 7-9 AND**	
				**Indeterminate Agreement**	Agreement**
Performance gaps	Importance	Unimportant	Equivocal	Important	Important – highest priority
CDS opportunities	Compatibility	Incompatible with clinical practice	Equivocal	Compatible with clinical practice	Compatible with clinical practice – highest priority
	Potential impact	Low potential impact	Equivocal	High potential impact	High potential impact – highest priority

*“No disagreement” implies either “agreement” or “indeterminate agreement”.

**While the RAND/UCLA Appropriateness Manual (RAM) only requires median ratings in the 7-9 range and *the absence of disagreement* to classify items as high priority, we used a higher bar, by requiring that each criterion exhibit statistical agreement. Items with indeterminate levels of agreement were not considered high priority.

Items that were classified as “equivocal” were not discussed after the first round of ratings. After the second round of rating the performance gaps, we moved forward the 8 highest rated gaps (+/- 4 gaps) that achieved “agreement” for having the panels consider CDS opportunities. This cut point was set to allow adequate time to discuss the one or more CDS opportunities for each performance gap in the third call, given the 90 minute phone call constraint.

From the two sets of ratings provided by experts on each panel, we compiled a list of high priority targets for CDS. Performance gaps that were highly rated on *importance* and for which the set of CDS tools or concepts was rated as being *compatible* with clinical practice and had a high *potential impact* on addressing the performance gap were designated high priority CDS targets.

## Selection of specialty-specific vs. condition-specific scope for expert panels

Constructing a panel first involves selecting the clinical domain that the panel will be asked to address. Conceptually, the focus of CDS prioritization could be on the breadth of problems within a single specialty or specialty subdomain (e.g., gastroenterology, including peptic ulcer disease, gastrointestinal malignancies, etc.) or it could focus on a condition from the perspective of the multiple specialties that treat the condition (e.g., viral hepatitis, including hepatologists, primary care physicians, infectious disease specialists, and transplant surgeons). Specialty-specific panels might be more likely to generate CDS priorities that are considered highly relevant within the specialty, however, this approach might reinforce the “silo” nature of medicine by failing to include the full scope of care provided for the conditions in question. A condition-specific approach, by contrast, provides an opportunity to bring together perspectives from a broad set of specialists to prioritize CDS that would best improve care for the condition, regardless of specialty. However, this approach would likely cover fewer conditions than the specialty-specific approach and it might have a higher risk of producing CDS priorities that miss the mark for specialties that are not strongly represented on the panel. Given these tradeoffs, we sought to include each kind of panel in pilot testing.

## Selection of expert panel members

Selection of panelists began with the recruitment of two co-chairs for each panel, one of whom was selected based on nationally-recognized leadership in the clinical domain and the other based on having expertise in CDS within the specialty. Co-chairs were selected in consultation with relevant specialty societies (such as the American College of Cardiology) and with staff of the AMA-PCPI, which is working with a broad range of specialties to set quality improvement objectives. After recruiting the panel co-chairs, we first consulted with them to refine the panel’s scope, setting bounds that would enable covering high-priority areas within the time available for the panel’s work. We then selected and recruited individual panelists based on their clinical expertise, community influence (i.e., in professional organizations for their specialty and serving on advisory panels related to quality of care, practice improvement, and/or use of health IT), and the diversity of settings in which they practice (to reflect both academic and community practice). For condition-specific panels, members were also selected to represent a balance of the specialties involved in caring for the conditions in question. For specialty-specific panels we also sought to include a relevant range of clinical sub-domains within the specialty.

The panel size was not fixed across panels. We selected approximately 14-17 members per panel to ensure that we would have a minimum of 9 panelists to complete the Delphi rating process after allowing for attrition.

## Scope and membership of panels for pilot testing the protocol

We selected four panels with different characteristics to maximize the amount of information learned during the pilot. To test variations on the definition of a panel’s area of clinical focus, we selected 1) one medical specialty (oncology), 2) one surgical specialty (orthopedic surgery), 3) one non-surgical procedural specialty (interventional cardiology), and 4) one primary care specialty (pediatrics). Key factors considered in selecting the focus, content and membership of the panels were as follows:

### Ensuring diversity of clinical workflows

The four panels were deliberately selected to represent a diverse set of workflows with known performance gaps potentially amenable to CDS. These workflows included: 1) Managing transitions between inpatient and ambulatory care settings (orthopedic surgery, interventional cardiology, oncology); 2) Care coordination with other specialists (oncology, interventional cardiology, pediatrics), 3) Care coordination during emergencies (interventional cardiology), 4) Selecting and implementing treatment protocols when the evidence base may be rapidly evolving (oncology); 5) Care provided by non-physician staff with specialized training (orthopedic surgery); 6) Workflows specific to different phases of illness (oncology); and 7) Long-term follow up and management (oncology, pediatrics) potentially facilitated by the use of registries (interventional cardiology, oncology).

### Variation in the use of EHRs and CDS

We also selected specialties that were known to be relatively advanced users of CDS (oncology) as well as those that were not known for having a high level of CDS development or EHR adoption (orthopedic surgery).

## Additional boundaries on clinical scope within specialty

Because the project was charged with developing and pilot testing a priority-setting protocol within a limited timeframe, we limited the clinical scope of each panel by selecting important specialty sub-domains, clinical conditions, or both, in consultation with the panel co-chairs. The considerations for each panel were as follows.

### Oncology

The oncology panel focused on medical oncology in recognition of the fact that medical oncologists are responsible for the largest share of all health expenditures for oncology. Also, given the large number of different cancers that could be addressed, we limited the focus to two of the most prevalent cancers, breast and colorectal cancer. While radiation oncologists and surgical oncologists have very different workflows that may define different CDS opportunities, we included two radiation oncologists and two surgeons on the panel to address the fact that medical oncologists commonly coordinate patient care with these other specialists. Thus, the oncology panel used a more condition-specific approach and it included input from multiple related specialties, but it still focused on care processes delivered by oncologists, such as the planning and administration of chemotherapy.

### Orthopedics

The scope of the orthopedics panel was confined to total hip and total knee replacement surgery, two of the most common procedures within the specialty. Many workflows and performance gaps associated with total joint replacement were also thought to be representative of those characteristic of other types of orthopedic surgery. We included a small number of spine and hand surgeons as well as a number of general orthopedic surgeons to understand areas where performance gaps and CDS opportunities might be common across these other areas.

### Pediatrics

While our pediatrics panel consisted entirely of pediatricians, a small number of panelists had expertise in selected pediatric clinical areas, such as allergy and behavioral health. The scope of the panel was restricted to pediatric conditions that were mostly treated in primary care settings.

### Interventional cardiology

For the interventional cardiology panel, we focused on percutaneous coronary intervention (PCI) both in the management of Acute Coronary Syndrome (ACS) as well as stable Coronary Artery Disease (CAD). Primary care-related performance gaps, such as the management of cholesterol levels, were not included. However, to make the panel more condition-specific rather than strictly specialty-focused, we included internists, interventional and non-interventional cardiologists, and electrophysiologists who had expertise related to the role of PCI and its coordination in managing ACS or stable CAD.

Members for each panel were selected based on the criteria noted above. We started by recruiting members who were known contributors to existing AMA-PCPI performance measurement panels—including those relating to breast cancer, colorectal cancer, and PCI. Many of the individuals had been nominated by their specialty organizations for developing performance measures through the AMA-PCPI. We then added clinical experts who were identified based on outreach to specialty organizations, use of key informants, and personal knowledge of experts by the project team. Because orthopedic surgeons were not heavily represented on any existing AMA-PCPI panels, we requested assistance from the American Academy of Orthopedic Surgeons (AAOS) and the North American Spine Society (NASS) to identify suitable experts, and particularly those individuals who had expertise with EHRs or clinical decision support. AAOS engaged in an open call to their membership while NASS recommended specific candidates.

## Results

The protocol was successfully implemented within each of the four specialty panels, and each produced lists of high priority targets amenable to CDS (Table 9). Each panel considered an initial set of 22 to 28 performance gaps, including numerous gaps nominated by individual panelists. Following the first stage of ratings, 6 to 15 gaps were classified as high priority across the four panels, with some panels endorsing gaps much more selectively than other panels. For example, orthopedic surgery and pediatrics panelists endorsed fewer than 21 percent and 39 percent of gaps, respectively, as highly important. Notably, less than half of the 43 clinical performance gaps that were rated “high priority” were based on quality measures, suggesting that clinical observation is an important source of data for determining priorities for CDS.

**Table 9:** Number of CDS targets rated high priority, by panel

**Panel**	**Performance gaps considered**	**“High priority” performance gaps**	**“High priority” performance gaps with effective and feasible CDS opportunities**
Oncology	22	15	14
Orthopedic surgery	28	6	3
Pediatrics	28	11	3
Interventional cardiology	23	11	4

Only the oncology panel found an abundance of highly effective and workflow-compatible CDS opportunities to address the high priority performance gaps. Fourteen of fifteen gaps were determined to be amenable to CDS compared to only 27, 36, and 50 percent of gaps for pediatrics, interventional cardiology, and orthopedic surgery, respectively. Nevertheless, all panels achieved consensus on at least 3 high priority targets for CDS. The complete set of rating results is available elsewhere.

All ratings were completed in a short timeframe with limited attrition. Preparation for and recruitment of the four panels was completed in 6 months and the panel protocol was implemented for all panels in only three months. Attrition by panelists was low despite a requirement for panelists to participate in all three teleconferences. Only two to three experts per panel withdrew during the course of the study. Our staged approach and use of webmeetings, were designed specifically to minimize participant burden and to enable rapid completion of the rating tasks and appeared to play a key role in the success of the pilot.

The need to implement the protocol in a short timeframe significantly limited the scope of our study. First, we were unable to conduct an extensive test of alternative frameworks and protocols for eliciting CDS targets. For example, we might have explored the use of a protocol that elicited very specific decision rules (rather than the broader construct of “targets”). We also might have incorporated specific workflows within which CDS might be optimally deployed in the rating tasks. These alternative frameworks may be worthy of additional study. Because of time constraints, we also did not allow panelists to nominate CDS opportunities beyond those developed by staff in conjunction with panel co-chairs. While a more comprehensive elicitation of available CDS interventions for each target might influence ratings, panelists were directed to rate the overall effectiveness and compatibility of the set of interventions for each performance gap as well as any other interventions that were not presented or discussed.

Rating the effectiveness and compatibility of CDS interventions appeared to be the most complex task for panelists. Many existing CDS interventions were described in the literature with varying levels of detail, and many CDS interventions rated by panelists were only concepts and were therefore difficult to fully specify. Developing standard templates for CDS interventions for use in future implementations of the protocol would help panelists have a common set of information on existing tools or tool concepts to facilitate the rating task. In addition, outreach to the vendor community to identify existing tools or tools in development might provide a more comprehensive set of CDS interventions to inform panelists’ ratings. Each of these preparatory activities highlights the importance of having an adequate number of skilled staff to conduct literature reviews and compile preliminary sets of rating items. The validity of the final set of high priority CDS targets could depend on the extent to which these initial activities are effective in identifying a comprehensive set of performance gaps and CDS interventions.

## Conclusion

The extent to which CMS’s EHR Incentive Programs will stimulate HIT-enabled quality improvement will depend to a large extent on the way in which meaningful use objectives are specified. We developed a conceptual framework and protocol for eliciting high priority CDS targets that are clinically important and that are amenable to CDS. These targets were elicited directly from specialists and reflect consensus recommendations following rating exercises and group discussions. While the targets are specific, they allow for a broad range of CDS interventions that can be used to address each performance gap. Such an approach recognizes specialists’ own experience and preferences for CDS while maintaining strong incentives for vendor innovation. CDS targets could be used to specify meaningful use objectives for the CMS EHR Incentive Programs or could play a role in other pay-for-performance programs.

## Endnotes

^a^This definition is a hybrid of the CDS definitions from the Health Information Management Systems Society (HIMSS) [39] and from CMS [36].

## Authors’ contributions

DB, JT, CD, and ES developed the conceptual framework and protocol, JT and DB drafted the manuscript. All authors read and approved the final manuscript.

## Pre-publication history

The pre-publication history for this paper can be accessed here:

http://www.biomedcentral.com/1472-6947/12/93/prepub
